# A model for delta rhythm fit to high-frequency cortical activity data

**DOI:** 10.1186/1471-2202-14-S1-P287

**Published:** 2013-07-08

**Authors:** Mark Reimers, Majid Mohajerani, Timothy H Murphy

**Affiliations:** 1 Dept Psychiatry, VCU, Richmond, VA 23298, USA; 2 Dept Psychiatry, UBC, Vancouver, BC, V6T 2A1, Canada

## Background

Rhythmic activity in the brain has been known since Berger's discovery of the alpha rhythm in the 1920's. Numerous mechanisms have been proposed for various rhythms but in the past half-century no consensus has been reached on the mechanism of any major rhythm. The recent development of high-throughput imaging methods enable us for the first time to rigorously and quantitatively test ideas about the dynamics of brain rhythms.

The aim of this project is to characterize the contributions of intrinsic dynamics of brain regions and network connections in generating the global dynamics of cortical activity using a mouse model.

## Methods

We have generated high-resolution data on neural activity over 40 mm^2 ^of mouse cortex by voltage-sensitive dyes, in both anesthetized and awake animals. We have instantiated current ideas about delta rhythm in models for the dynamics of such activity and we measure the fit of these models quantitatively in predicting this data, thus shedding light on the relative contributions of these processes in the delta rhythm. We include in the model intrinsic regional oscillations, thalamic input, and communication between cortical regions. We specify the form of the model and then estimate the parameters by fitting the dynamical behavior to high-resolution time series of cortical activity in mouse cortex. We try to estimate the relative contributions of each of the major components of the model to the fluctuations.

## Results

We show that a potassium-current mechanism for intrinsic oscillations does in fact fit the data well, and we estimate some of the effective connectivity between different cortical regions under anesthesia.

